# SPRC Suppresses Experimental Periodontitis by Modulating Th17/Treg Imbalance

**DOI:** 10.3389/fbioe.2021.737334

**Published:** 2022-01-11

**Authors:** Qian Peng, Bingkun Zhao, Jie Lin, Haixia Liu, Rong Zhou, Dongmei Lan, Chao Yao, Shaohua Cong, Shen Tao, Yizhun Zhu, Raorao Wang, Shengcai Qi

**Affiliations:** ^1^ Department of Stomatology, Shanghai Tenth People’s Hospital, Tongji University School of Medicine, Shanghai, China; ^2^ Hubei No. 3 People’s Hospital of Jianghan University, Wuhan, China; ^3^ Pharmacy Department, Minda Hospital of Hubei Minzu University, Enshi, China; ^4^ Medical College of Anhui University of Science and Technology, Huainan, China; ^5^ Jiading Central Hospital, Shanghai University of Medicine and Health Sciences, Shanghai, China; ^6^ The First People’s Hospital of KunShan, Kunshan, China; ^7^ State Key Laboratory of Quality Research in Chinese Medicine and School of Pharmacy, Macau University of Science and Technology, Taipa, Macao SAR, China; ^8^ Department of Prothodontics, Shanghai Key Laboratory of Craniomaxillofacial Development and Diseases, Shanghai Stomatological Hospital, Fudan University, Shanghai, China

**Keywords:** S-propargyl-cysteine, periodontitis, Th17 cells, regulatory T cells, ERK/CREB signalling pathway

## Abstract

**Object:** The aims of the study were to explore the protective effects of S-propargyl-cysteine (SPRC) on periodontitis and to determine the underlying mechanisms.

**Methods:** A rat periodontitis model was constructed by injecting LPS and SPRC (0, 25, and 50 mg/kg/d) was administered intraperitoneally. H2S and CSE level were detected. The alveolar bone level was evaluated by micro-CT, HE staining and methylene blue staining analysis. Inflammation-related factors, Treg and Th17 cells were detected by immunohistochemistry, RT-PCR, immunofluorescence, Western blot and flow cytometry. Phosphorylation levels of ERK1/2 and CREB were analysed.

**Results:** The administration of SPRC significantly increased the expression of CSE in the gingival tissue and the concentration of endogenous H2S in the peripheral blood. Simultaneously, SPRC significantly inhibited the resorption of alveolar bone based on the H&E staining, micro-CT and methylene blue staining analysis. Compared with the periodontitis group, the levels of IL-17A, IL-10 were downregulated and IL-6,TGF-β1 were upregulated in the SPRC groups. In the SPRC group, the percentage of TH17 cells and the expression of ROR-γt were downregulated, while the percentage of Tregs and the expression of Foxp3 were upregulated accompanied with inhibition of phosphorylation ERK1/2 and CREB.

**Conclusion:** SPRC can prevent the progression of periodontitis by regulating the Th17/Treg balance by inhibition of the ERK/CREB signalling pathway.

## Introduction

Periodontitis (PD) is a prevalent chronic inflammatory diseases and caused by infection of bacteria on the surface of teeth,which is characterized by destruction of soft and hard periodontal tissue (Graves et al., 2006). It has been confirmed to be a risk of several systemic diseases, including Parkinson’s, diabetes, respiratory diseases, and cardiovascular diseases (Slots, 2017). The pathogenesis of periodontitis and the development of disease has much relation with a combination of dysbiosis of the microbiome and the dysregulated inflammatory response of the host. During the progression of chronic PD, periodontal pathogens initiate the occurrence of PD, while the host immune response determines the outcome of the disease (Cekici et al., 2014; Hajishengallis, 2015).

The response to periodontal bacteria from host immune, especially CD4^+^ T cell mediating host immune response, play an essential role in PD progression. Retinoic acid-related orphan receptor gamma transcription (RORγt) factor, a predominantly proinflammatory mediator, is expressed on Th17 cells and act a crucial role in immune-mediated diseases and tissue injury by producing the characteristic cytokine IL-17 (Lee, 2018). IL-17 stimulates adjacent immune cells to produce inflammatory factors such as IL-6, and upregulates the expression level of activator of nuclear factor-kappa B ligand (RANKL) in osteoblast/stromal cells to accelerate inflammatory progression. It was reported that Th17 cell-related cytokines cantribute to periodontal breakdown, inhibiting IL-17 alleviated periodontal breakdown (Bunte and Beikler, 2019). Regulatory T cell (Treg) expression of Forkhead box p3 (Foxp3), which has a regulatory function and suppresses effector T cell function, exerts an anti-inflammatory role by secreting anti-inflammatory cytokines (such as transforming growth factor-β (TGF-β) and IL-10) to help balance of immune homeostasis which can alleviate immune-mediated tissue injury (Sakaguchi, 2003; Lee, 2018; Deng et al., 2019). Thus, Treg cell-related cytokines is negative relate to periodontal breakdown in periodontitis patients and inhibit the function of Treg cells in exacerbated periodontal lesions. In inflammation site, the activiation of Th17 cells can be inhibited by the migration of Tregs. These results suggest that imbalance in Th17/Treg cells has an important function in the pathogenesis of periodontitis (Rose et al., 2015). Therefore, regulating the TH17/Treg balance is a useful target for developing new methods for treating periodontitis.

Hydrogen sulfide (H2S) is a gasotransmitters *in vivo* which is synthesized from L-cysteine and/or L-homocysteine by cystathionine β-synthase (CBS), cystathionine γ-lyase (CSE) and 3-mercaptopyruvate sulfurtransferase (3-MST) activiation (Szabo, 2018). Significant research of H2S has been made in recent years to a better understaing of physiological role of in immune regulation. Exogenous sources of H2S, NaHS, and GYY4137 (a slow H2S-releasing agent) showed significant anti-inflammatory properties in inflammatory diseases (Rose et al., 2015). NaHS, an H2S provider, significantly improved the proportions of Th17/Treg and RORγt/Foxp3 expression and played a therapeutic role in treating rheumatoid arthritis, multiple sclerosis, and psoriasis vulgaris (Etesam et al., 2016; Wu et al., 2016b; Ma et al., 2018). Endogenous H2S plays an immunomodulatory role by promoting polarization of Treg cell and inhibiting Th17 cell through the coupling pathway. The endogenous H2S produced by activation of T cells is used as an autocrine or paracrine enhancer for cell activation. Moreover, H2S upregulates the expression of Tetl and Tet2 to promote the differentiation and stability of Treg cells, CD4 T-Cell. Endogenous Cystathionine γ Lyase-Hydrogen Sulfide can attenuates hypertension by sulfhydrating liver kinase B1 to promote T regulatory cells (Rose et al., 2015; Wu et al., 2016a).

S-propargyl-cysteine (SPRC, also known as ZYZ-802), a novel water-soluble modulator of endogenous H2S, exerts protective effects against various inflammation by CSE activiation (Wen et al., 2018). However, SPRC in other inflammatory diseases, such as PD, is still unclear. Considering that SPRC has anti-inflammatory and immunoregulatory properties, we tried to investigate whether SPRC could prevent the development of PD and its mechanisms in the present study.

## Materials and Methods

### Animals and Rat Periodontitis Model Construction

**FIGURE 1:**
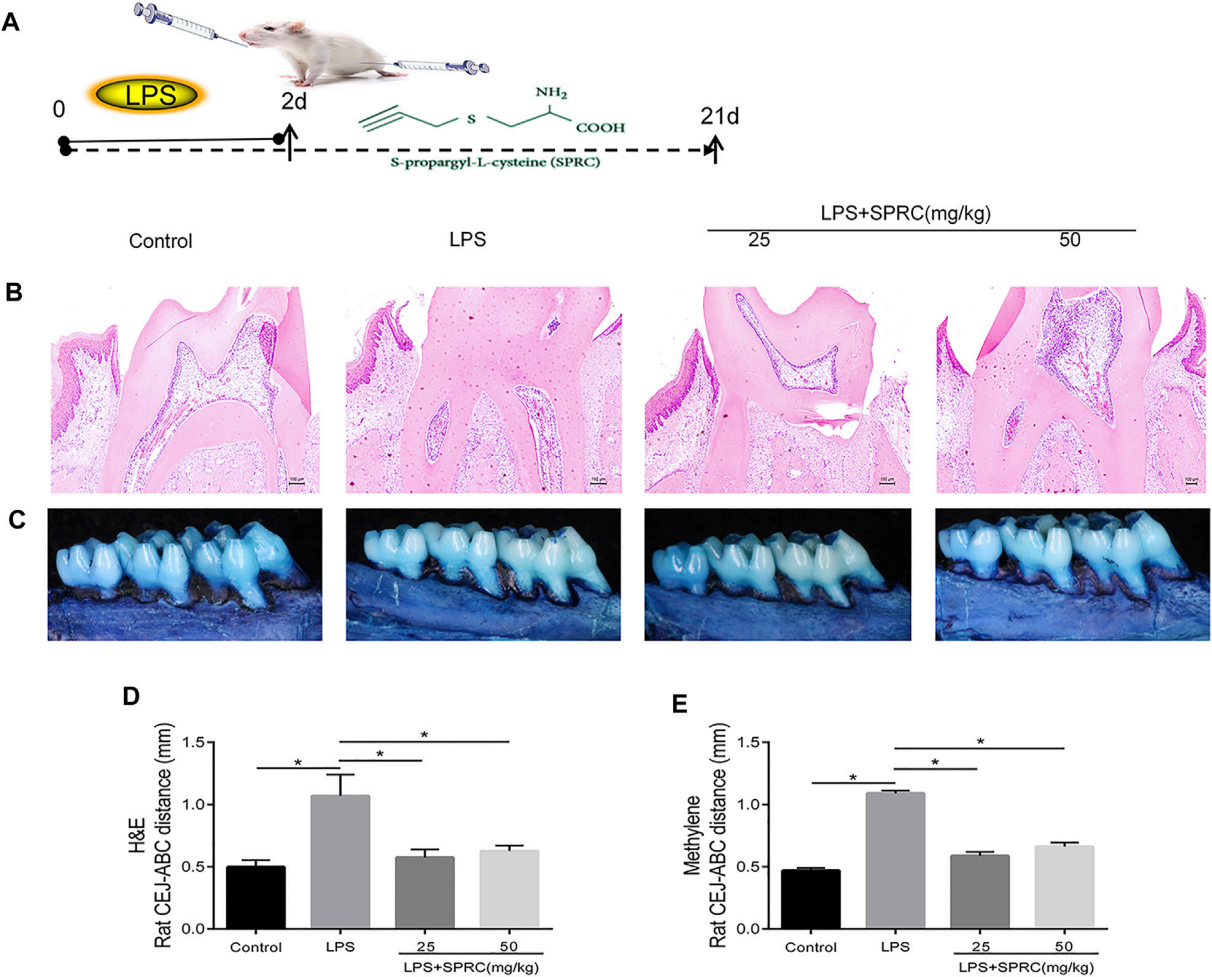
Effect of SPRC on bone resorption. The crest height between the (CEJ) and the alveolar bone crest (ABC). **(A)**Schema chart **(B)** H&E staining of bone resorption of maxilla sections (×200 magnification scale bar = 100 μm) **(C)** Methylene staining of bone resorption of left maxilla, **(D)** H&E staining data quantitative analysis **(E)** Methylene staining data quantitative analysis. Specifically, measurements were performed on the first molars (at 3 sites), second molars (at 2 sites), and third molars (at 1 sites), red line indicate CEJ-ABC distance (Data are shown as the means ± SD from eight animals *p < 0.05 vs. LPS group).

40 Male Sprague-Dawley rats (200–230 g) specimen were received from Shanghai Victoria Laboratory Animals (Shanghai, China) and kept in a standard laboratory for animals. All experimental equipment and procedures were examined and confirmed by the Animal Care and Use Committee Shanghai Tenth People’s Hospital, Tongji University School of Medicine. They were anaesthetized with 2% pentobarbitital sodium (0.2 mL/100g), intragingival injection of 2 μl of LPS(*Salmonella typhimurium*, Sigma, United Kingdom) (10 μg/μl/d) between the first molar and the second molar induced periodontitis once a day for the first 2 days, SPRC was administered by intraperitoneal injection everyday for 21 days in the model. The injection was made at the mesolateral side of the interdental papilla on both sides of the maxillary with a Hamilton microsyringe (Hamilton, Switzerland). It was performed slowly, and the needle was kept in place for seconds after injection to guarantee that LPS was not lost after extraction. To evaluate periodontitis lesions, rats were sacrificed at 21 days. Rats were divided into the following experimental groups (*n* = 10 for each group) (Figure 1A).

−Control group: rats received a single intragingival injection of saline solution with an identical surgical procedure as that described above, and 2 mL of saline was administered by intraperitoneal injection (There were two died when intraperitoneal injection for the anaesthetization).
−LPS group: rats were subjected to LPS-induced periodontitis as described above, and 2 mL of saline was administered by intraperitoneal injection (There was two died, one is for intraperitoneal injection, the other maybe is for the inflammation).
−LPS + 25 (mg/kg) SPRC group: rats were subjected to LPS-induced periodontitis as described above, and SPRC (25 mg/kg) was administered by intraperitoneal (There was two died, one is for the anaesthetization, the other maybe is for intraperitoneal injection).
−LPS + 50 mg/kg SPRC group: similar to the LPS + 25SPRC group, the concentration of SPRC was changed to 50 mg/kg.

### Blood Sample Collection and H2S Detection

Blood samples from the intraorbital region of all the rats were collected after 21 days induction of periodontitis. Four Collected blood samples from each group were prepared for flow cytometry analysis of TH17 and Tregs. The other 4 collected blood samples were used to determined the hydrogen sulfide content in serum samples by a micro H2S Content Assay Kit (BC2055 Solarbio, China) according to the manufacturer’s instructions.

### Flow Cytometric Analysis of Treg and Th17 Cells From the Peripheral Blood

PBMCs (7 × 10^5^ cells) were stained with certain antibodies against L/D-FVS510, CD45-FITC, CD4-PE, CD25-BV421 (all from Biolegend, United States) to analyse the Treg frequency, and FOXP3-APC (EB). The impacted cells were run with BD FACSCalibur and BD FACSAria III flow cytometers (BD Biosciences, United States). Using positive gates and gating controls by Fluorescence minus one (FMO) controls. For Th17 cell analysis, cells were incubated with phorbol-12-myristate-13-acetate (PMA) (50 ng/mL), ionomycin (1 μg/mL), and monesin (1.7 μg/mL; Sigma-Aldrich, United States) at 37°C for 6 h to activate T cells and stimulate the accumulation of intracellular IL-17. Then, the cells were stained with specific antibodies against L/D-FVS510, CD45-FITC, CD4-PE (all from Biolegend, San Diego, California, United States), and IL-17A-APC (EB). The FACSCalibur flow cytometer (BD Biosciences) was used to analyse the stained cells.

### Maxilla and Soft Tissue Sample Collection and Treatment

The Right maxilla and soft tissue from eight samples in each group were collected and fixed in 4% paraformaldehyde. The four fixed maxilla were scanned and analysed by micro-CT using an Inveon Micro CT system, and all the eight samples then decalcified in 10% EDTA (EDTA 100 g, NaOH 11 g, NaHPO4 6 g, NaH2PO4 9 g, ddH2O, Mix with 900 mL magnetic mixer, with ddH2O capacity to 1000 mL and PH to 7.2–7.4 and 4°C). After dehydration, the maxillae were embedded in paraffin for paraffin section. The paraffin sections were stained with haematoxylin and eosin (H&E). Meanwhile, immunofluorescence (IF) staining and immunohistochemical staining were proceed in the next experiments. The left maxilla and gingival tissue from eight samples in each group were collected. The maxilla was stained by 1% methylene blue and then detected using the vernier caliper. The gingival tissue was saved at −80°C for mRNA and protein extraction.

### Micro-CT Analysis

The right maxilla was selected as the tissue specimen. After fixing it in 4% paraformaldehyde for 24 h, the specimens were placed on the scanning bed parallel to the occlusal surface of the molar crown. Then, micro-CT (μCT100, Seanco Medical, Bassersdorf, Switzerland) scanning was conducted (pixel resolution = 10 μm, 50 kV, 500 A).

**FIGURE 2:**
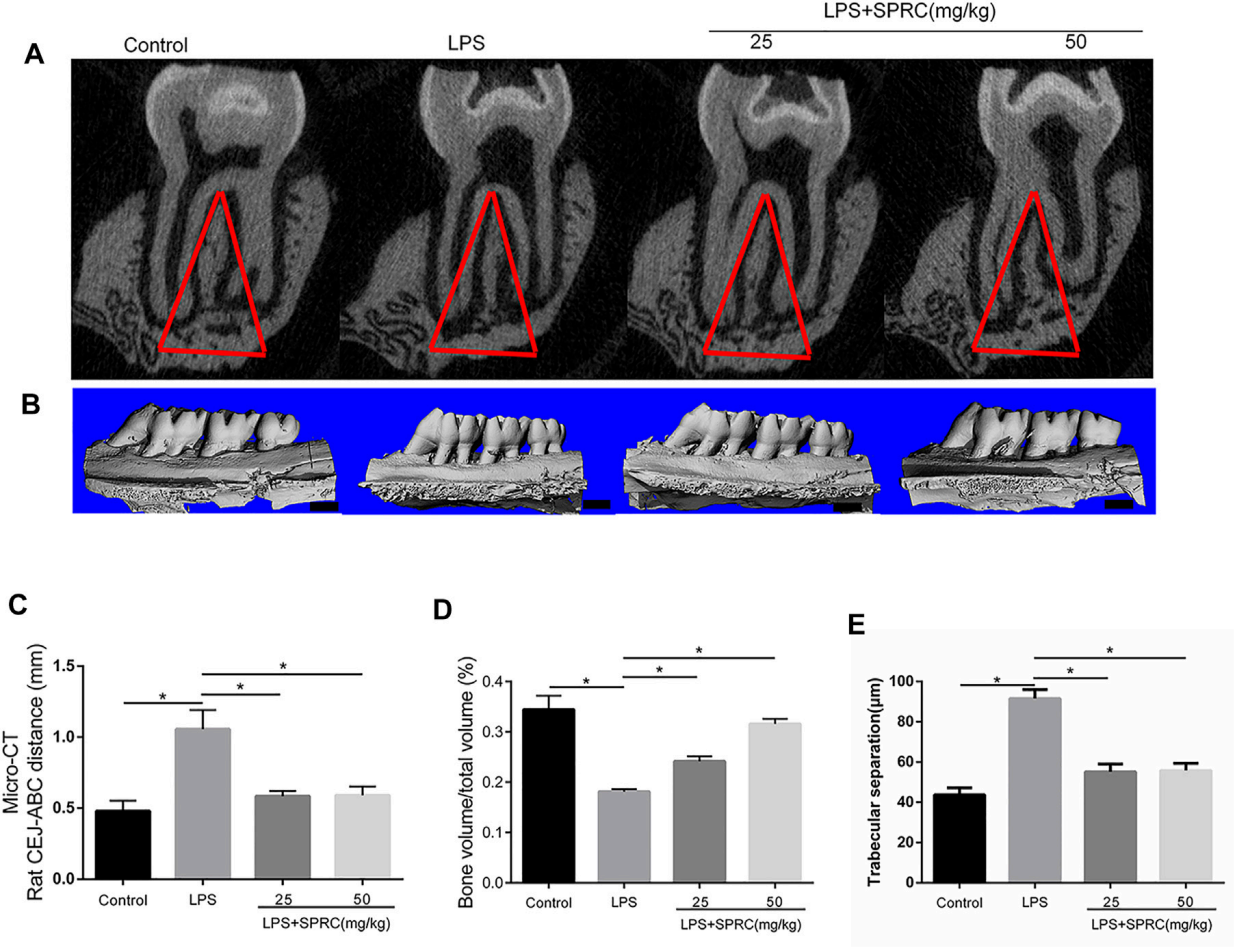
Bone resorption of right maxilla were assessed by micro-CT. **(A)** Coronal plane of the area under the second molar bifurcation (2D picture)red line indicate region of interest (ROI), **(B)** Sagittal 3D images **(C)** CEJ-ABC distance **(D)** Micro-CT evaluation of the bone volume fraction of the residual alveolar bone in the maxilla (scale bar = 100 μM), **(E)** Microstructural parameters of the trabecular bone in the maxilla, trabecular separation (Tb.Sp) (Data are shown as the means ± SD from 4 animals vs. LPS group).

The original data were reconstructed by the NRecon software (Skyscan, Kontich, Belgium) and transferred to the CTAnalyser and CTVox software (Skyscan, Kontich, Belgium) for further analysis. Semi-automatically select the triangular area below the fork root of the second molar root was selected as region of interest (ROI).

(Figure 2A, Red line area). The cancellous bone in the ROI was selected and measured by two investigators thrice. The outcome measures included: bone surface/bone volume (BS/BV), and trabecular separation (Tb.Sp). Alveolar bone crest height was determined measurements were taken on all molars of the right palatal. Specifically, measurements were performed on the first molars (at 3 sites), second molars (at 2 sites), and third molars (at 1 sites). Altogether, 6 sites were evaluated in each animal. The alveolar bone crest level was assessed as the distance from cementoenamel junction to the alveolar bone crest (CEJ-ABC). The CEJ-ABC distance at all aforementioned sites was analyzed by a blinded investigator three times at different time points. Mean values from the three measurements were calculated for further analyses.

### Immunohistochemical and IF Staining

The deparaffinized sections were subjected to heat mediated antigen retrieval, and blocked in H2O2 for half an hour, followed by PBS with 5% BSA and 0.2% Triton X‐100 at normal temperature for half an hour, and then stained overnight with primary anti-CSE(1:100, Santa Cruz Biotechnology, Inc SC-365381), anti-IL-17A (1:500; Proteintech13082-1-AP), anti-IL-6 (1:100, Abcam, ab208113), anti-TGF-β1 (1:100, Abcam, ab215715), and anti-IL-10 (1:100, Abcam, ab225820). Tissues were incubated with secondary antibody (HRP‐Donkey Anti‐Goat IgG, Proteintech after rinsing with PBS for 15 min, Cat#SA00001‐3) at normal temperature. Sections were washed and colors were developed with DAB (3,3′‐diaminobenzidine). Finally, the haematoxylin was used and a Leica DM6 B fluorescence microscope was used to capture images.

For IF staining, the slices were incubated with anti-ROR-γt (1:1000; Abcam, ab219496) or anti-Foxp3 (1:1000; Abcam, ab22510) antibodies at 4°C overnight after blocking (Thermo Scientific). Then, the slices were incubated in the presence of Alexa Fluor 594 or Alexa Fluor 488-labelled secondary antibodies after washing with PBS three times. The nucleus was visualized with DAPI and images were captured with a CarlZeiss LSM710 fluorescence microscope.

### Western Blot Analysis

Four Gingival tissues (ranging from the mesial site of the first molar to the mesial site of the third molar at the palatal faces) in each group diced into fragments and homogenized in sodium dodecyl sulfate sample buffer containing proteinase and phosphatase inhibitors. THP-1-derived macrophages were lysed with RIPA lysis buffer (Beyotime, China). Collected supernatants, and measured using a BCA assay kit for protein concentrations. The total protein samples were separated by sodium dodecyl sulfate-polyacrylamide gel electrophoresis and transferred to nitrocellulose membranes (Amersham Pharmacia Biotech, United Kingdom). After blocking with 5% BSA for 2 h, incubated membranes at 4°C overnight with primary antibody. All antibodies were diluted to working concentrations with antibody dilution buffer (U3510 sigma-aidrich). CSE (1:100, Santa Cruz Biotechnology, SC-365381), IL-17A (1:500, Proteintech, 13082-1-AP), IL-6 (1:100, Abcam, ab208113), TGF-β1 (1:100, Abcam, ab215715), and IL-10 (1:100, Abcam, ab225820), Foxp3 (1:1000, Abcam, ab215206), ROR-γt (1:1000, Abcam, ab219496), ERK1/2 (1:1000, Cell Signaling Technology-4695), P-ERK1/2 (1:1000, Cell Signaling Technology-4370), CREB (1:1000, Abcam, ab32515), and P-CREB (1:1000, Abcam, ab32096), GAPDH (1:10,000, Kangcheng, China). The following day, after washing by PBST for 3 times, each washing time is 10 min, membranes were incubated with second antibodies for 1 h. Specific bands were visualized by Tanon‐5200 Multi Chemiluminescent System (Tanon). Grayscale values were measured and counted using Image J software.

### Real Time Polymerase Chain Reaction Analysis

Total RNA was extracted from 4 gingival tissues (ranging from the mesial site of the first molar to the mesial site of the third molar at the palatal faces) in each group by TRIzol (Thermo Fisher Scientific, United States). cDNAs were reversely transcribed with the PrimeScript RT Master Mix (Takara, Cat#RR036A) and subjected for RT‐qPCR using specific primers. Listed the sequences of the forward and reverse primers in Supplementary Table S1 on line.

### Statistical Analysis

Experimental data repeated at least three times are presented as the mean ± SD. Student’s t-test was used to analyse the differences between two groups. One-way ANOVA Student-Newman-Keuls method was used to compare significant distinction among three or four groups by using SPSS version 20.0 software (IBM, Armonk, NY, United States). *p < 0.05* was considered as statistically significant.

## Results

### Effect of SPRC on Bone Resorption

Methylene stereomicroscopy, H&E and micro-CT results showed bone resorption in periodontitis rats. The distance from the CEJ to the ABC was quantitated as periodontal bone loss under a methylene stereomicroscope. The distance between both sides of the ABC was quantitated by H&E staining. Compared with the control group, periodontal bone resorption was significantly increased in the LPS group by methylene stereomicroscopy and H&E (Figures 1B–E, *p < 0.05), which showed that the rat periodontitis model was successfully constructed. In the LPS + 25SPRC group and LPS + 50SPRC group, periodontal bone resorption was significantly lower than that in the LPS group by methylene stereomicroscopy and H&E (Figures 1B–E, *p < 0.05). Additionally, compared with the control group, the second molar bifurcation area (2D picture) under alveolar resorption increased in the LPS group, 3D reconstruction by micro-CT also showed that the distance from CEJ to ABC increased in the LPS group (Figures 2A–C, *p < 0.05), and the BV/TV of the LPS group was significantly lower than that in the control group (Figure 2D, *p < 0.05), and the Tb.Sp of the LPS group was significantly higher than that in the control group (Figure 2E, *p < 0.05), which showed that the rat periodontitis model was successfully constructed. Meaningfully, the second molar bifurcation area (2D picture) under alveolar bone resorption was obviously inferior in the LPS + 25SPRC group and LPS + 50SPRC group than that in the LPS group (Figure 2A), 3D reconstruction by micro-CT showed that the distance of the CEJ-ABC was obviously shorter in the LPS + 25SPRC group and LPS + 50SPRC group than that in the LPS group (Figures 2B,C, *p < 0.05), and the BV/TV was significantly increased after SPRC treatment (in both the LPS + 25SPRC group and LPS + 50SPRC group) (Figure 2D, *p < 0.05). The Tb.Sp was significantly decreased after SPRC treatment (in both the LPS + 25SPRC group and LPS + 50SPRC group) (Figure 2E, *p < 0.05). All of these results showed that SPRC inhibited LPS-induced periodontal bone resorption in the rat periodontitis model.

### Effect of SPRC on Plasma H2S Concentration and CSE Expression in Gingival Tissue

**FIGURE 3:**
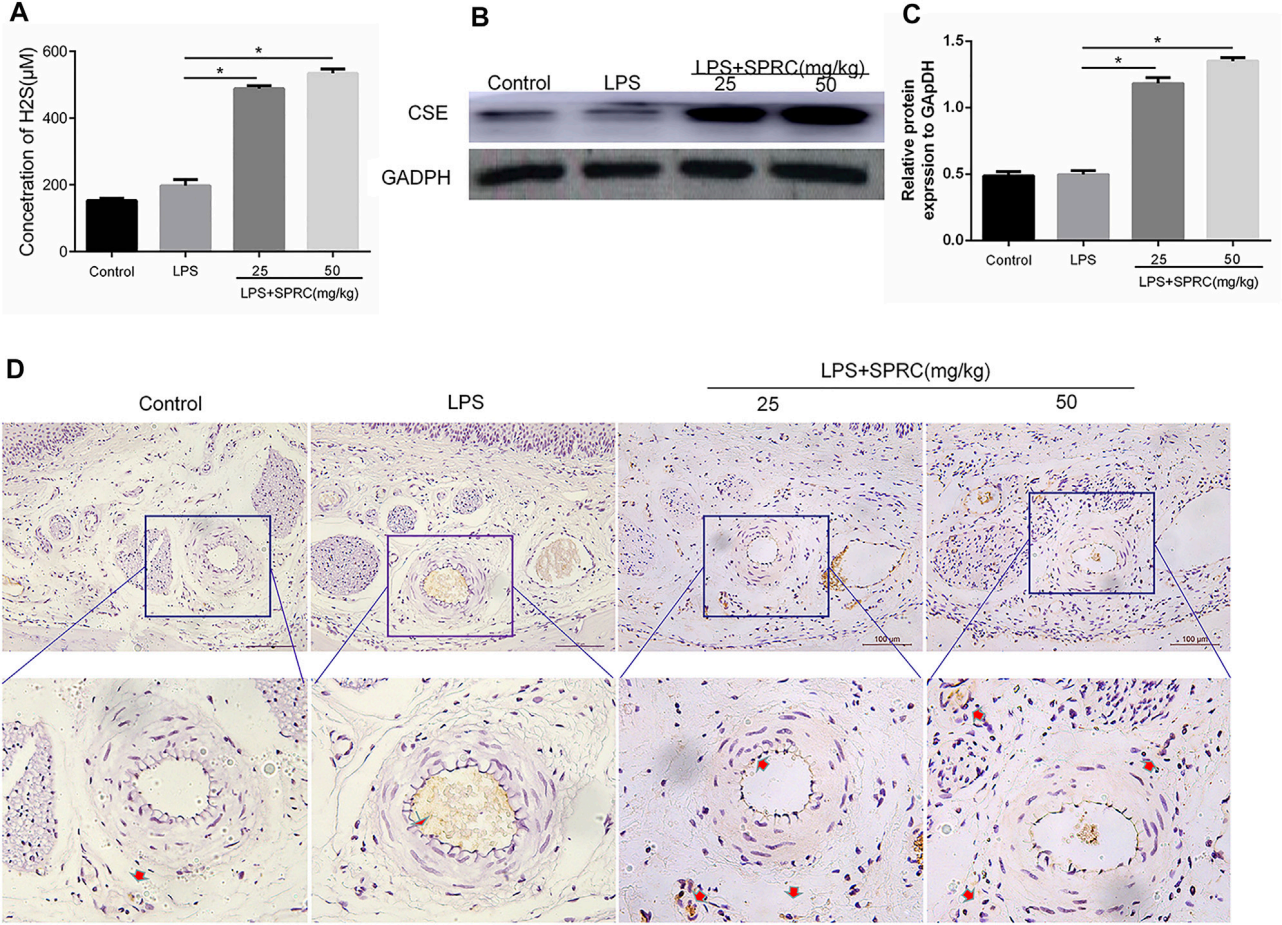
Plasma H2S concentration and CSE expression level in gingival tissue. **(A)** The content of H2S in peripheral blood after SPRC applied for 3 weeks. **(B)** Western blot detection of CSE enzyme expression level in gingival tissue. **(C)** Quantitative analysis of western blot results. **(D)** Immunohistochemical localization detection of CSE enzyme expression in lingual gingival (perivascular tissue), above (×200 magnification scale bar = 100 μm), lower (×400 magnification) (Data are shown as the means ± SD from 4 animals, *p < 0.05 vs. LPS group).

To identify whether endogenous H2S changed with SPRC treatment, the levels of H2S in plasma were first examined after 21 days of rat periodontitis. There were no significant differences between the control group and periodontitis group in terms of the levels of H2S in plasma. The levels of H2S were obviously higher in the plasma in the LPS + 25SPRC group and LPS + 50SPRC group than in the LPS group (Figure 3A, *p < 0.05). CSE is a key H2S-producing enzyme in gingival tissue. The Western blot and IHC results showed trends similar to those of H2S in gingival tissue (Figures 3B–D).

### Flow Cytometry Analyses of Th17/Treg Cells in Peripheral Blood

**FIGURE 4:**
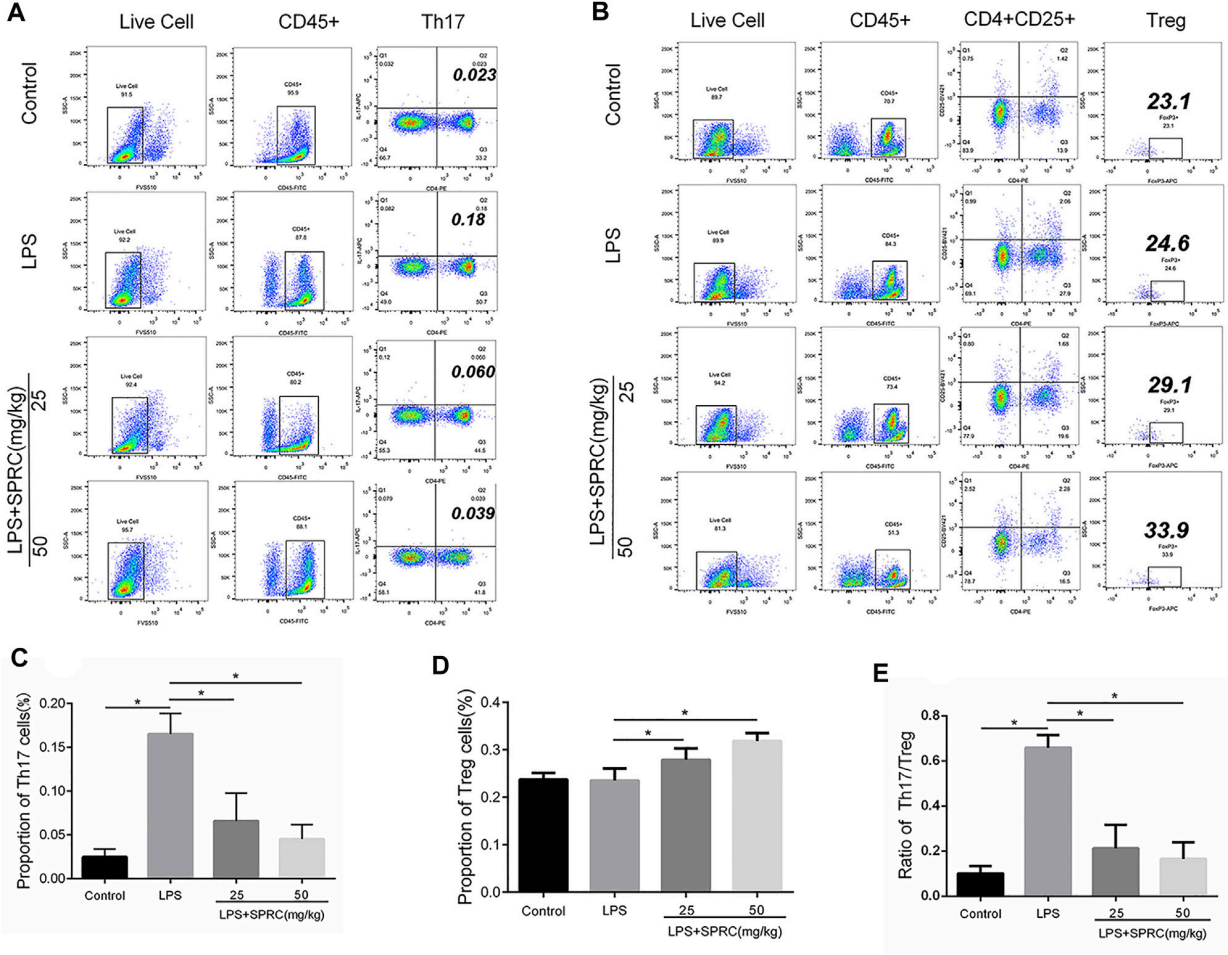
Proportion of Th17 cells and Treg cells. The ratio of Th17/Treg in rats’ peripheral blood. Live/die (L/D, CD45, CD4, CD25, IL-17A, and Foxp3, was analyzed by flow cytometry. **(A)** Dot plot in the upper separate representation Live Cell, CD45^+^, right quadrant represents CD4+IL-17A+(Th17cell) in gated CD45 + cell. **(B)** Dot plot in the upper separate representation Live Cell, CD45^+^, right quadrant represents CD4^+^CD25+in gated CD45 + cell, Foxp3+(Treg cell)+in gated CD4^+^CD25 + cell **(C)**: Histogram expression of CD4+IL-17A+(Th17cell) percentages in gated CD45 + cell, **(D)** Histogram expression of Foxp3+(Treg cell) percentages in gated CD4^+^CD25 + cell, **(E)** Histogram expression of Th17/Treg ratio. Data are shown as the means ± SD from 4 animals. *p < 0.05 vs. LPS group.

Th17/Treg imbalance has been implicated in the pathogenesis of periodontitis. The frequencies of Th17 and Treg cells in peripheral blood were determined by flow cytometry in our study. The results showed that the percentage of Th17 cells significantly increased in the LPS groups (0.1650% ± 0.02380) compared to the control group (0.02475% ± 0.009069) (*p < 0.05). The percentage of Th17 cells was significantly reduced in both the LPS + 25SPRC group (0.06567% ± 0.03188) and LPS + 50SPRC group (0.04500% ± 0.01657) compared with the LPS group (0.02475% ± 0.009069) (Figures 4A,C, *p < 0.05). The percentage of Treg cells was not significantly different between the LPS group (0.2350% ± 0.02536) and the control group (0.2370% ± 0.01400). The Treg cells were showed as the mean percentage (%), respectively, for the LPS + 25SPRC group (0.2788% ± 0.02392) and LPS + 50SPRC group (0.3180% ± 0.01709), which were significantly higher than those in the LPS group (0.2350% ± 0.02536) (Figures 4B,D, *p < 0.05). Compared to the control group, the Th17/Treg ratio was particularly increased in the LPS groups, which showed a similar trend to that in human periodontitis. Most importantly, the ratio of Th17/Treg was rescued in the LPS + 25SPRC group (0.2123% ± 0.1040) and LPS + 50SPRC group (0.1656% ± 0.07420), both of which were significantly lower than that in the LPS group (0.6592% ± 0.05622) (Figure 4E, *p < 0.05). These results indicated that SPRC regulated the balance of Th17/Treg cells in peripheral blood by inhibiting Th17 cells and promoting Treg cell differentiation in a rat periodontitis model.

### Effect of SPRC on Th17 and Treg Cell-Related Cytokines

**FIGURE 5:**
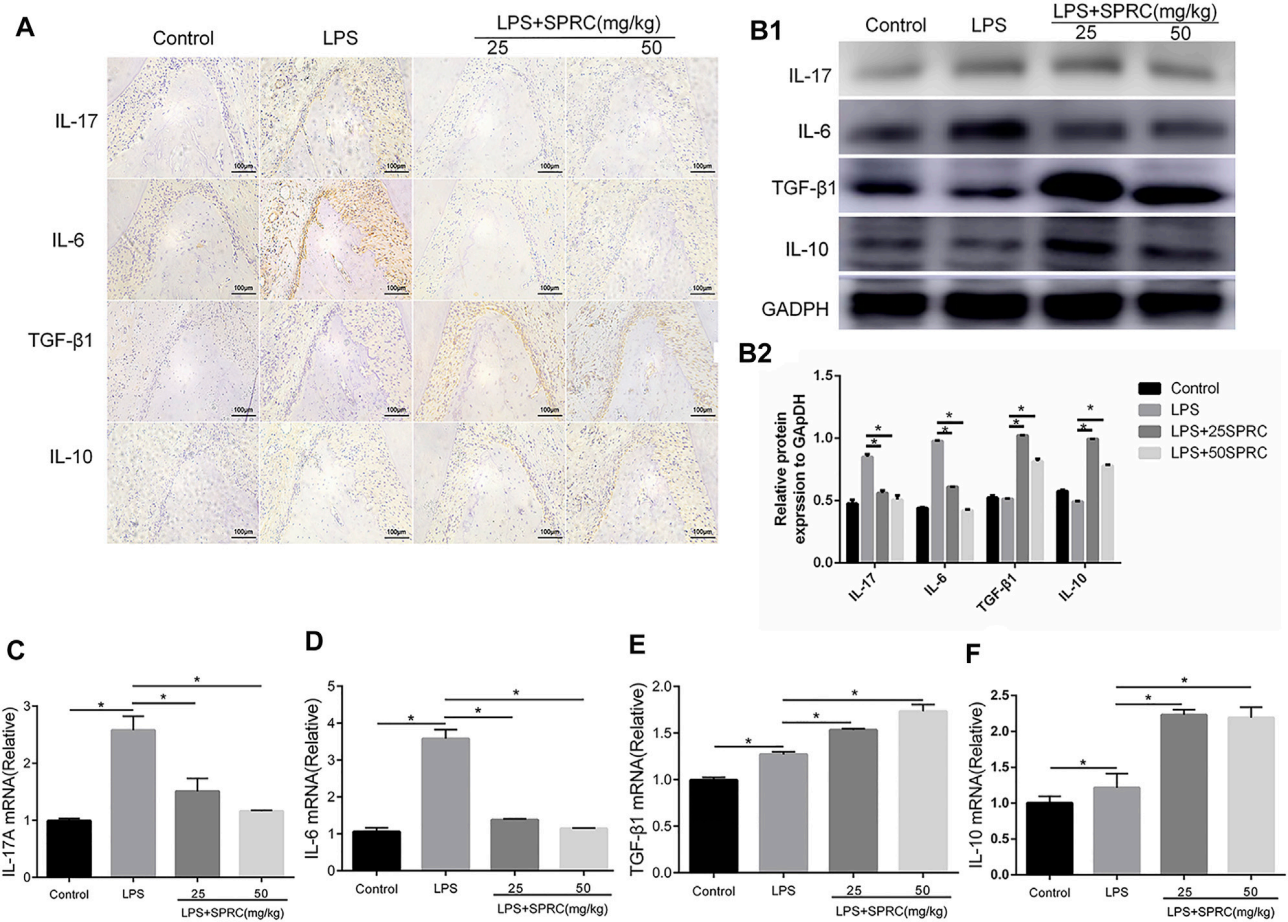
Effect of SPRC on Th17/Treg cell–related cytokines. **(A)** Immunohistochemical localization assays for IL-17A,IL-6, IL-10 and TGF-β1 protein in gingival tissue (×200magnification scale bar = 100 μm), **(B1)** Western blot assays for IL-17A, IL-6, IL-10 and TGF-β1 protein in gingival tissue. **(B2)** Quantitative analysis of western blot results., **(C–F)** Histogram expression of RT-PCR assays for IL-17A,IL-6, IL-10 and TGF-β1 mRNA in gingival tissue, Data are shown as the means ± SD from 4 animals. *p < 0.05 vs. LPS group.

Th17 and Treg cell-related cytokines (IL-17, IL-6, IL-10, and TGF-β) were detected by IHC, Western blot and RT-PCR. The results showed that the protein and mRNA expression of IL-17 and IL-6 in gingival tissue was significantly upregulated in the LPS group compared with the control group. The protein and mRNA expression of IL-17 and IL-6 was obviously downregulated in the LPS+25SPRC and LPS+50SPRC groups, which was almost the same as the control group. Additionally, there were no significant differences between the LPS group and the control group in the protein and mRNA expression of IL-10 and TGF-β. The protein and mRNA expression of IL-10 and TGF-β was much higher in the LPS+25SPRC group and LPS+50SPRC group than in the LPS group (Figure 5). All of these results show that SPRC alleviated the progression of periodontitis by inhibiting proinflammatory cytokine (IL-17 and IL-6) expression and promoting anti-inflammatory cytokine (IL-10 and TGF-β) expression in a rat periodontitis model.

### Effect of SPRC on the Expression of ROR-γt and FoxP3 in Gingival Tissues

**FIGURE 6:**
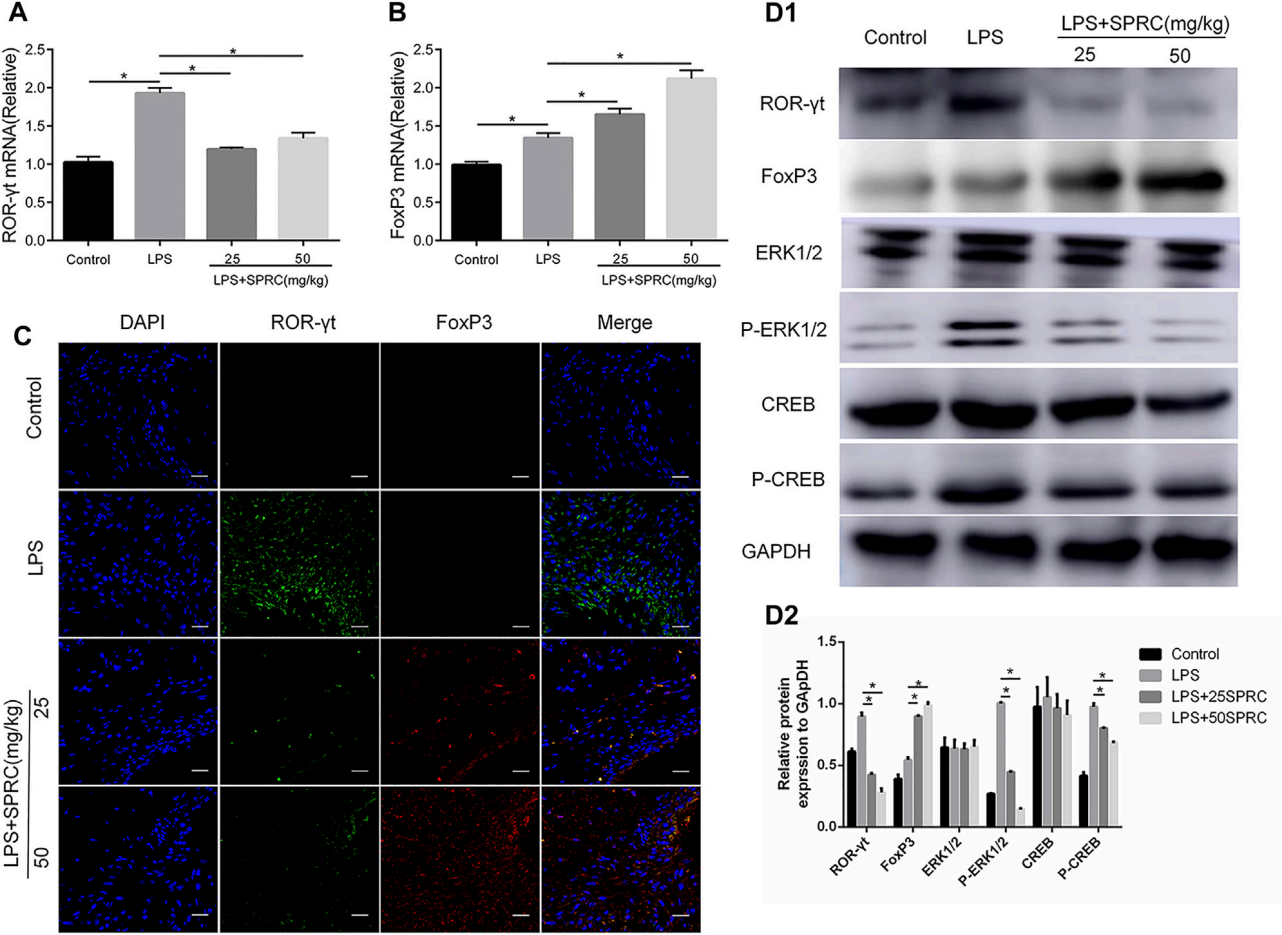
Effect of SPRC on expression of ROR-γt, FoxP3 and ERK1/2, CREB phosphorylation in gingival tissues, **(A,B)** Histogram expression of RT-PCR assays for ROR-γt and FoxP3 mRNA in gingival tissue ,**(C)** Confocal microscopy results. ROR-γt (Alexa Fluor 488, green), FoxP3 (Alexa Fluor 638, red) and DAPI nuclear staining merged channels (×600 magnification scale bar = 20 μm). **(D1)** Western blot assays for ROR-γt, FoxP3,ERK1/2, CREB protein express and ERK1/2, CREB phosphorylation levels in gingival tissue. **(D2)** Quantitative analysis of western blot results. Data are shown as the means ± SD from 4 animals. *p < 0.05 vs. LPS group.

The specific transcription factors RORγτ and FoxP3 were detected by RT-PCR and IF to further analyse the Th17 and Treg percentages in gingival tissues. The mRNA level of RORγτ was significantly increased in the LPS group compared to the control group. Compared with the LPS group, the mRNA level of RORγτ was significantly inhibited in the LPS+25SPRC group and the LPS+50SPRC group (Figure 6A, *p < 0.05). Compared with the control group, the expression of Foxp3 mRNA was significantly decreased in the LPS group. Meaningfully, the expression of Foxp3 mRNA was obviously higher in the LPS+25SPRC group and LPS+50SPRC group than in the LPS group (Figure 6B, *p < 0.05). IF analysis showed that there was significantly higher expression of ROR-γt (green) in the LPS group than in the control group. Furthermore, ROR-γt (green) was mainly expressed in the periodontal ligament. After SPRC treatment (the LPS+25SPRC group and LPS+50SPRC group), ROR-γt (green) was significantly weaker in gingival tissues than in the LPS group. In contrast, the expression of FoxP3 (red) was not different between the LPS group and the control group. FoxP3 (red) in gingival tissues was significantly stronger in the LPS+25SPRC group and LPS+50SPRC group than in the LPS group. Sections were counterstained with DAPI (blue) (Figure 6C). The protein levels of RORγτ and FoxP3 showed the same results by Western blot (Figures 6D1,D2).

### SPRC Inhibits the CREB/ERK1/2 Signalling Pathway in a Rat Periodontitis Model

The present study found that the p-ERK1/2 and p-CREB levels were upregulated in rat periodontitis tissues in the LPS group. However, SPRC notably inhibited phosphorylated CREB and ERK1/2 at doses of 25 and 50 mg/kg compared with the LPS group (Figures 6D1,D2). The results showed that SPRC may inhibit the CREB/ERK1/2 signalling pathway in the rat periodontitis model.

## Discussion

Inflammation in PD is a multiple process that involves the secretion of innate and adaptive immune cells and their molecules, including B cells, CD4^+^ T cells, and NK cells. Recently, endogenous H2S is confirmed to play an important role in inflammation and immune regulation in many diseases (Dilek et al., 2020). Treatment with H2S-releasing medicines reduced the level of inflammatory process associated with intragingival LPS injection and had a positive effect on LPS-induced experimental rat periodontitis (Gugliandolo et al., 2018). Therefore, we hypothesize that SPRC, a modulator of endogenous H2S, may play a protective role in periodontitis.

H2S has roused considerable interest as a potential signalling molecule in cellular systems, body temperature, metabolic levels and the induction of periodontal ligament cell apoptosis (Cen et al., 2016). The role of endogenous H2S has generated great controversy. H2S upregulated RANKL to promote osteoclast differentiation. H2S deficient mice displayed an osteoporotic phenotype (Behera et al., 2018). Research found that the expression of CBS and CSE for H2S production was upregulated, while there was no difference in the H2S level in periodontitis. Endogenous H2S is necessary to maintain osteogenic differentiation of human periodontal ligament cells by activating the Wnt/b-catenin signalling cascade (Cen et al., 2016). Although many hydrogen sulfide protective effects have been elucidated, they cannot be used for clinical therapy because of their gaseous nature. SPRC, a slow H2S-releasing drug, provides cysteine, a substrate of CSE, and possesses acceptable pharmacokinetic properties in rats (Wen and Zhu, 2015). SPRC is recognized as the substrate for endogenous H2S synthesis via CSE catalysis and can increase CSE and H2S production, which is consistent with our findings (Figure 3). SPRC exert protective effects in cardiovascular disease, neurodegenerative disease, cancer, and many inflammatory disease (Wen et al., 2018). However, the effects of SPRC on periodontitis and the underlying mechanism remain unclear. Therefore, this study investigated the role and possible mechanisms of SPRC in LPS-induced periodontitis.

Bone resorption and inflammatory factors were observed in the LPS group (Figures 1, 2). In the SPRC groups, SPRC upregulated the levels of H2S and CSE in gingival tissue. Moreover, SPRC inhibited bone resorption, as determined by methylene blue staining, HE staining and micro-CT (Figures 1, 2). All of these results showed that SPRC protected bone in LPS-induced rat periodontitis *in vivo*.

The discovery of Th17 cells and Treg cells further complements the pathogenesis of periodontal disease and provides a new idea for the treatment of periodontitis (Gao et al., 2017). Endogenous H2S induce anti-inflammatory effects by modulate various immune cell functions, including Th17 and Treg functions (Yang et al., 2018; Lin et al., 2019). More importantly, T cells express the enzymes of CBS and CSE, which help generation of H2S(Kaur et al., 2015; Wang et al., 2020). H2S is a necessary mediator for Foxp3+ Treg cell differentiation. Loss of Treg cells in CBS-deficient mice resulted in early lethality and immune cell infiltration into various tissues. In terms of mechanism, H2S can contribute to Tet-mediated activaion of DNA demethylation to facilitate Treg cell–specific hypomethylation inducing Treg cell stability (Yang et al., 2015). H2S serves as a costimulator in modulating T cell activation. In our study, the increased percentage of Th17 cells were found, while the percentage of Treg cells was not changed in LPS-induced experimental periodontitis. In the SPRC groups, the percentage and number of Th17 cells were significantly reduced, and the percentage and number of Tregs were significantly upregulated in the blood (Figure 4). IL-17, IL-6, IL-10 and TGF-β from Th17 and Treg cells are considered cytokines related to PD. Th17 cytokines (IL-17 and IL-6) promote the activation of T cells and stimulate cell adhesion molecules on the surface of macrophages to induce inflammation and aggravate bone destruction. IL-10 and TGF-β secreted from Treg cells inhibit the proliferation of memory T cells, thus inhibiting the occurrence of inflammation (Wang et al., 2014; Lee, 2018; Cafferata et al., 2020). In our study, the mRNA and protein levels of IL-17 and IL-6 (Th17 cytokines) were upregulated in the LPS-induced group but downregulated in the SPRC groups in gingival tissue. The mRNA and protein levels of IL-10 and TGF-β in gingival tissue were downregulated in the LPS-induced group but upregulated in the SPRC groups in gingival tissue (Figure 5). These results suggest that SPRC effectively modulates the Th17/Treg balance, thus contributing to protection against periodontitis by maintaining the balance of proinflammatory and anti-inflammatory cytokine expression and reducing alveolar bone loss.

To further explore the molecular mechanism by which SPRC regulates the function and differentiation of Th17 and Treg cells in periodontitis, the ERK1/2 signalling pathway was analysed in this study. Hydrogen sulfide can suppressing PHD2/HIF-1α/MAPK signaling pathway to inhibits cigarette smoke-induced inflammation and injury in alveolar epithelial cells and NaHS is also a H2S provider, which can reduces ERK activation in psoriatic lesions even with a dramatic decrease in its nuclear localization (Mirandola et al., 2011; Guan et al., 2020). Previous research found that the transcription factor cyclic AMP-responsive element binding protein (CREB) can moderate the balance between Th17 and Treg cells (Liu et al., 2013; Lv et al., 2018). CREB, the downstream of the ERK1/2 signalling pathway, can be activated by TcR stimulation in thymocytes and T cells (Muthusamy and Leiden, 1998). Research showed that CREB is an essencial role in ROR factors directing IL-17 expression level (Wang et al., 2017). CREB can bind to conserved noncoding sequence (CNS) 2 region in the Foxp3 gene locus and activate its promoter (Ogawa et al., 2014). Computational analysis revealed that CREB may cooperate with RORγt in controlling IL-17 transcription. Additionally, activating CREB will reduce Th17 cell differentiation *in vitro* and *in vivo* (Symons and Ouyang, 2017). Overexpression of CREB significantly reduce IL-17 expression in Th17 cells. In our study, the expression of RORγt was increased, and the expression of Foxp3 was decreased in the LPS group. SPRC upregulated Foxp3 expression and downregulated RORγt in gingival tissue. More importantly, the levels of CREB and ERK1/2 phosphorylation in the SPRC treatment group were decreased compared with those in the LPS group (Figure 6). These results suggest that SPRC can inhibit CREB and ERK1/2 phosphorylation, finally achieving regulation of the Th17/Treg balance through inhibition of RORγt expression and promotion of Foxp3 expression in chronic periodontitis.

**FIGURE 7:**
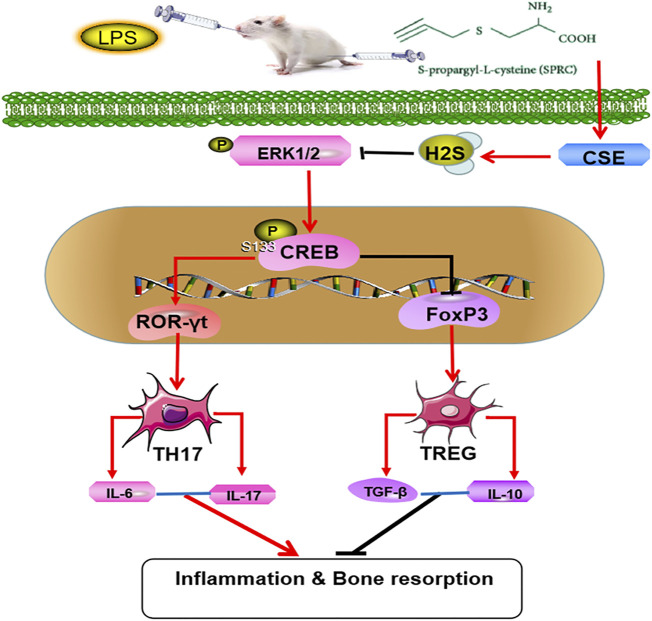
Mechanisms of SPRC in alleviating periodontitis,SPRC prevent the rat chronic periodontitis through synthesis of endogenous H2S by CSE catalysis. Endogenous H2S inhibited the activation and proliferation of Th17 cells and promoted the activation and proliferation of Treg cells through inhibiting ERK1/2 and CREB phosphorylation. (Red lines indicate promotion, black lines indicate inhibition).

In summary, our research suggests that SPRC can inhibit the progression of periodontitis by regulating the Th17/Treg balance through ERK1/2/CREB signalling pathway (Figure 7). However, the relationship between SPRC, Th17/Treg and the ERK1/2/CREB pathway needs further exploration, and more clinical trials should be carried out before SPRC is allowed for patient use.

## Data Availability

The original contributions presented in the study are included in the article/Supplementary Material, further inquiries can be directed to the corresponding authors.

## Supplementary Material

Supplementary Table S1 The sequences of primers for qRT-PCR analysis.
