# Supplementary material for: SPRC Suppresses Experimental Periodontitis by Modulating Th17/Treg Imbalance
Source: Front Bioeng Biotechnol. 2022 Jan 11;9:737334. doi: 10.3389/fbioe.2021.737334 (PMC8787365; doi:10.3389/fbioe.2021.737334)
Supplement: Supplementary file 1 [file Table1.DOCX]

| **Gene** |  | **Primer sequence（5'-3'）** |
| --- | --- | --- |
| **GAPDH** | **Forward primer** | **CAGGGCTGCCTTCTCTTGT** |
|  | **Reverse primer** | **TCCCGTTGATGACCAGCTTC** |
| **TGF-β1** | **Forward primer** | **AGAGCCCTGGATACCAACTA** |
|  | **Reverse primer** | **CAACCCAGGTCCTTCCTAAAGC** |
| **IL-10** | **Forward primer** | **TGGGAAGTGGGTGCAGTTAT** |
|  | **Reverse primer** | **GCTCAGCACTGCTATGTTGC** |
| **IL-6** | **Forward primer** | **GGTTTGCCGAGTAGACCTCA** |
|  | **Reverse primer** | **GTGGCTAAGGACCAAGACCA** |
| **IL-17A** | **Forward primer** | CAACCTGAACATCCATAACC |
|  | **Reverse primer** | GTCGGCTCTCCATAGTCT |
| **ROR-γt** | **Forward primer** | **CAGCGCTCCAACATCTTCT** |
|  | **Reverse primer** | **CCACATCTCCCACATGGAC** |
| **FoxP3** | **Forward primer** | **CAGTACCCCCAAATTCCTGC** |
|  | **Reverse primer** | **GCTGAAGACGTGTGCATCCTA** |
