# Supplementary figures and images for: SPRC Suppresses Experimental Periodontitis by Modulating Th17/Treg Imbalance
Source: Front Bioeng Biotechnol. 2022 Jan 11;9:737334. doi: 10.3389/fbioe.2021.737334 (PMC8787365; doi:10.3389/fbioe.2021.737334)

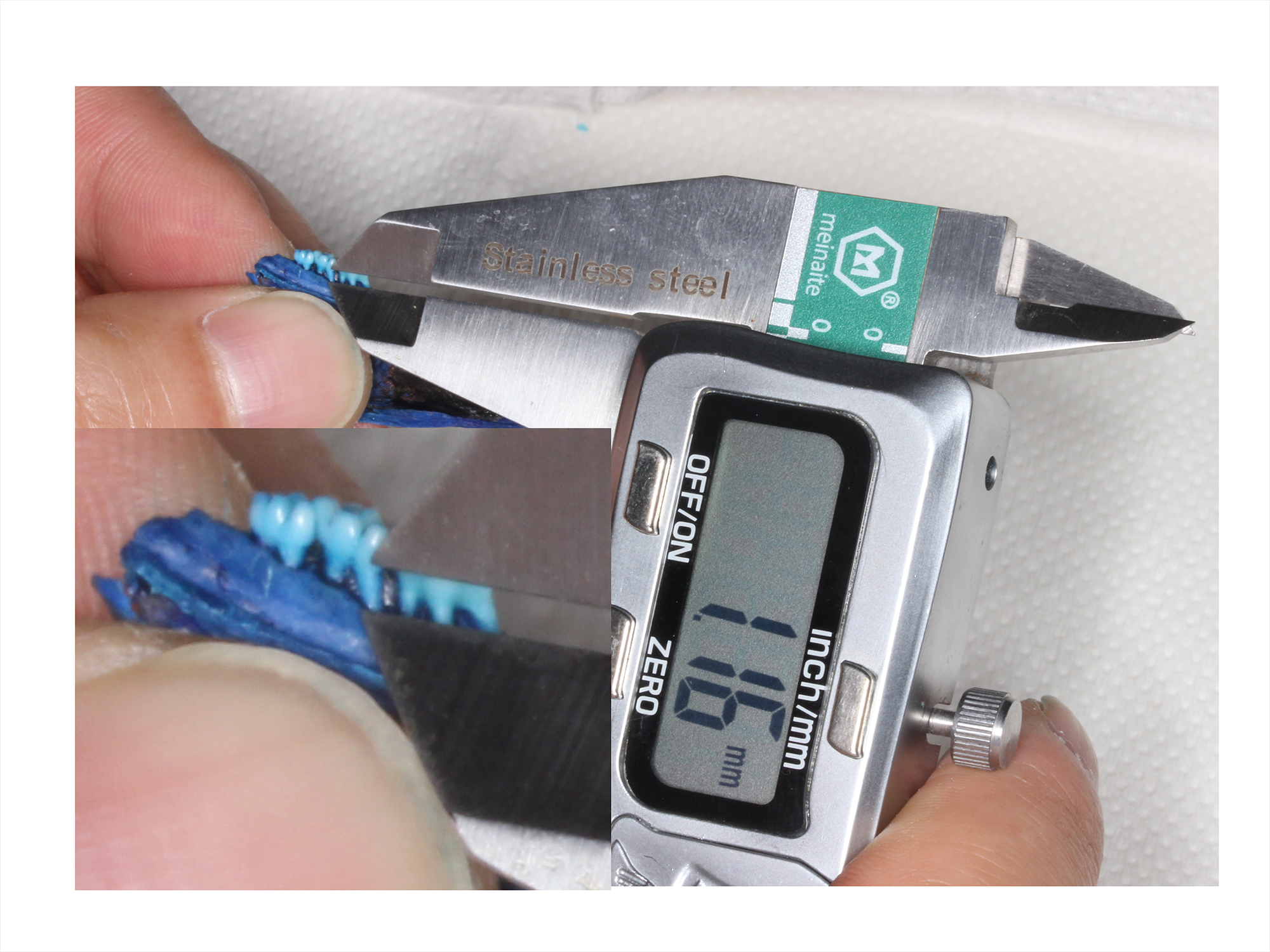

Supplement: Supplementary file 2 [file Image2.TIF]

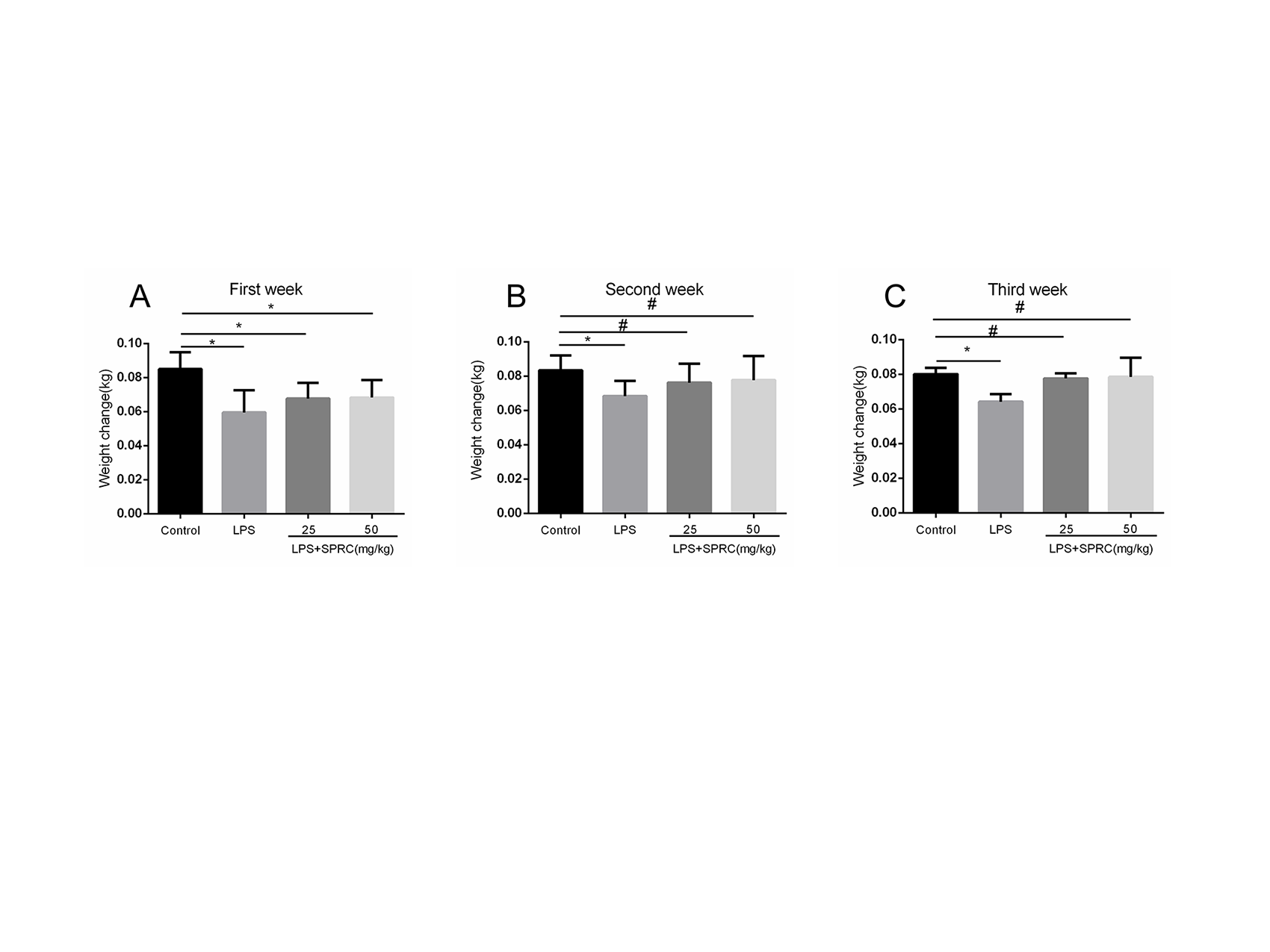

Supplement: Supplementary file 3 [file Image1.TIF]
